# Reverse Buffering Effects of Active Coping on Suicidal Ideation in Bullied Adolescents: Age Cohort Differences

**DOI:** 10.3390/bs15121619

**Published:** 2025-11-25

**Authors:** Sichen Liu, Qing Xiong, Ya Gao, Le Wang, Quanlei Yu

**Affiliations:** 1 Key Laboratory of Adolescent Cyberpsychology and Behavior (CCNU), Ministry of Education, Central China Normal University, No. 152 Luoyu Road, Hongshan District, Wuhan 430079, China; 2 Key Laboratory of Human Development and Mental Health of Hubei Province, School of Psychology, Central China Normal University, No. 152 Luoyu Road, Hongshan District, Wuhan 430079, China; 3 School of Sociology and Humanities, Jiangxi University of Finance and Economics, No. 168 East Shuanggang Road, Nanchang 330013, China

**Keywords:** Bullying Victimization, Suicidal Ideation, Active coping, age cohort

## Abstract

Previous research indicates that bullying victimization is a key predictor of adolescent suicidal ideation. From the perspective of the Integrated Motivational–Volitional (IMV) model, active coping strategies may buffer the feelings of defeat and humiliation caused by bullying victimization, thereby mitigating the emergence of suicidal ideation. However, this buffering effect weakens as the severity of bullying increases, reflecting a reverse buffering pattern. Moreover, due to developmental differences in the effectiveness of coping under varying levels of bullying severity, this moderating effect is further influenced by age cohort. To investigate the moderating role of active coping strategies and the moderating effect of age cohort (early-to-middle adolescents vs. late-age adolescents) in the bullying victimization–suicidal ideation relationship, we collected data from 3227 Chinese students, measuring bullying victimization, suicidal ideation, active coping strategies, age and other demographic variables. The results revealed that (a) bullying victimization was significantly and positively associated with suicidal ideation and (b) active coping significantly buffered this relationship; however, the buffering effect weakened as bullying severity increased, and (c) the reverse buffering effect of active coping was significant among early-to-middle-aged adolescents but not among late-age adolescents. This study offers important implications for designing targeted prevention and intervention strategies to reduce suicidal ideation among youth exposed to bullying.

## 1. Introduction

Adolescent mental health has become a global concern, with suicide rates exhibiting a disturbing upward trend (World Health Organization, 2025). Suicidal ideation—defined as thoughts, intentions, or plans to harm oneself or end one’s life—is a critical precondition to suicidal behavior and a strong predictor of suicide attempts and deaths among youth (O’Connor & Kirtley, 2018; Sveticic & Leo, 2012; Klonsky et al., 2016). Among its key risk factors, bullying victimization—intentional, repeated aggression within a power imbalance—has consistently emerged as a significant predictor (Gaffney et al., 2019; Meltzer et al., 2011; Klomek et al., 2007). However, the strength of this association varies, indicating the presence of moderating factors (Holt et al., 2015; J. Sun et al., 2024). Drawing from the Integrated Motivational–Volitional (IMV) model, active coping strategies may buffer the adverse emotional impacts of bullying, reducing the likelihood of suicidal ideation (Labelle et al., 2013; Yin et al., 2017). Yet, due to differences in the severity of bullying victimization across developmental stages, the effectiveness of coping strategies may vary between early-to-middle and late-age adolescents (Glenn et al., 2020). This study applies the IMV model to investigate the moderating role of active coping and age cohort, aiming to clarify the mechanisms linking bullying victimization and suicidal ideation and to inform targeted prevention efforts.

### 1.1. The Relationship Between Bullying Victimization and Suicidal Ideation

Bullying victimization is a widespread issue among adolescents (Ariani et al., 2025), with recent research indicating alarmingly high prevalence rates. A meta-analysis reported that approximately 36% of adolescents had experienced some form of bullying victimization (Modecki et al., 2014). This sustained and distressing experience—often characterized by repeated exposure to failure, humiliation, and power imbalance—can result in significant physical, psychological, social, and academic consequences for victims (J. Chen et al., 2025). Adolescents subjected to bullying frequently may suffer from low self-esteem, social withdrawal, peer rejection, and feelings of worthlessness. These experiences are commonly accompanied by increased levels of anxiety and stress, which not only impair psychological well-being but also reduce academic motivation and performance (Liao et al., 2025). In more severe cases, the consequences of bullying victimization can escalate to suicidal ideation and suicidal behavior (Bauman et al., 2013; Kim et al., 2009; Kim & Leventhal, 2008; Kerns et al., 2025). Notably, one study reported that individuals who were bullied in childhood are 3.75 times more likely to attempt suicide over their lifetime than those who were not bullied. (Meltzer et al., 2011). Given the potentially devastating effects, the relationship between bullying victimization and suicidal ideation has become a central focus in adolescent mental health research (Baiden & Tadeo, 2020; Holt et al., 2015; Modecki et al., 2014), prompting the development of theoretical models to explain the emergence of suicidal ideation.

According to the Integrated Motivational–Volitional (IMV) model (O’Connor & Kirtley, 2018; Comtois et al., 2025), the development of suicide unfolds across three sequential phases. The pre-motivational phase encompasses an individual’s background vulnerabilities—genetic, cognitive, biological, and environmental—that heighten susceptibility to suicidal thoughts and behaviors. When these vulnerabilities are activated by adverse life events, the individual transitions into the motivational phase, in which psychological mechanisms contribute to the emergence of suicidal ideation. A central mechanism during this phase is the progression from feelings of defeat and humiliation to a pervasive sense of entrapment—the belief that there is no escape from one’s suffering (Comtois et al., 2025). If suicidal ideation intensifies or persists, the individual may move into the volitional phase, where factors such as impulsivity and acquired capability for suicide increase the risk of acting on suicidal thoughts. School bullying, as a form of chronic social trauma, often inflicts repeated psychological harm, including failure, shame, and social exclusion (J. Sun et al., 2024). If these distressing experiences remain unresolved and victims perceive no viable means of escape, they may develop a deep sense of entrapment (O’Connor & Kirtley, 2018). When this sense reaches a tipping point, suicide may be perceived as the only solution, thereby giving rise to suicidal ideation. Thus, continuous bullying may accelerate a person’s psychological transition from distress to entrapment and ultimately to suicidal ideation.

### 1.2. The Potential Moderating Role of Active Coping

Extensive research has demonstrated a robust association between bullying victimization and suicidal ideation (Bauman et al., 2013; Cao et al., 2023; Kim & Leventhal, 2008; Klomek et al., 2010), while this relationship shows variability across studies (Jiang et al., 2025; J. Sun et al., 2024). Drawing on the IMV model, which conceptualizes suicide as a progression through three distinct phases, the motivational phase is the one most closely associated with suicidal ideation. Within this phase, Threat-to-Self Moderators (TSMs) and Motivational Moderators (MMs), which influence the development of suicidal ideation, are identified. The former influences the transition from feelings of defeat or humiliation to entrapment, while the latter can either amplify or attenuate the progression from entrapment to suicidal ideation. When these moderator functions as protective factors, they can reduce individuals’ feelings of isolation and hopelessness, help them recognize alternatives to suicide, and foster a more hopeful outlook. Importantly, coping is explicitly identified as both a prototypical TSM and an MM (Comtois et al., 2025). *Coping* refers to the dynamic process by which individuals manage or adapt to stress by modifying their behavioral, emotional, cognitive, or physiological responses, depending on changing demands and circumstances (Compas et al., 2001). Previous studies have commonly classified coping strategies into active coping and passive coping (Guadalupe & DeShong, 2025; Hinch & Sirois, 2024). Active coping refers to constructive efforts to confront or mitigate stressors, and passive coping is characterized by avoidance, denial, or disengagement (Compas et al., 1999). Compared to passive coping, active coping strategies more effectively alleviate the negative emotions induced by stressors (Compas et al., 2001) and also promote positive emotions that counteract conditions like anxiety and depression (Hansen et al., 2012; Lai et al., 2023; P. Sun et al., 2019; S. Xie et al., 2022). Therefore, bullying victims who employ active coping strategies may experience greater emotional resilience and a diminished risk of developing suicidal ideation. 

Given the capacity of active coping strategies to mitigate the negative impact of stressors, they have been explicitly identified as both threat-to-self moderators and motivational moderators in the pathway from bullying victimization to suicidal ideation. This theoretical framework highlights their dual role in disrupting both the transition from defeat to entrapment and from entrapment to suicidal ideation. In the motivational phase, active coping serves as a threat-to-self moderator by enabling individuals to proactively manage or diminish stressors (Liang et al., 2020), thereby impeding the development of a perceived inescapable situation. For example, active coping strategies such as problem-solving to address bullying directly or cognitive restructuring to reframe distressing thoughts can lessen the feelings of repeated failure and humiliation that contribute to entrapment (Labelle et al., 2013). Simultaneously, active coping also functions as a motivational moderator by attenuating the escalation from entrapment to suicidal ideation. Even when individuals feel trapped, active coping like emotion regulation, seeking social support, and identifying alternative solutions can offer psychological relief, foster hope, and reduce the belief that suicide is the only escape (Liang et al., 2020; Yin et al., 2017). Additionally, meaning in life has been conceptualized as a form of active coping (Türk et al., 2024), and adolescents with a strong sense of purpose are less likely to develop suicidal ideation, even under conditions of bullying (Henry et al., 2014). Thus, active coping may buffer the association between bullying victimization and suicidal ideation by interrupting both critical transitions within the IMV model’s motivational phase.

While the protective potential of active coping is underscored, this buffering effect may diminish as the severity of bullying intensifies—known as the reverse buffering effect. Previous studies found that the capacity of most active coping strategies, such as help-seeking and cognitive restructuring, is effective under moderate stress, and becomes less impactful in high-severity bullying contexts (Nixon et al., 2020). Based on the IMV model (O’Connor & Kirtley, 2018), severe bullying leads to the accumulation of defeat, humiliation, and helplessness, which can result in a deeply rooted sense of psychological entrapment. In such situations, the victim’s self-concept deteriorates further, and active coping strategies may no longer suffice to alleviate the emotional turmoil (X. Zhang et al., 2012). Moreover, when these coping strategies fail to produce tangible improvements, they may paradoxically amplify feelings of hopelessness, reinforcing the belief that no viable escape exists, except through suicide (Chou et al., 2018). Thus,

**Hypothesis** **1.** *Active coping strategies may moderate the relationship between bullying victimization and suicidal ideation, showing a reverse buffering effect such that their protective function would weaken as bullying severity increases.*

### 1.3. The Difference Among Age Cohorts

The moderating effect of active coping on the association between bullying victimization and suicidal ideation may be further influenced by age cohort. A three-level meta-analysis of anti-bullying interventions found that the effectiveness of these interventions, including active coping strategies, significantly decreases in late adolescence (15–19 years) (Yeager et al., 2019). This decline in efficacy has been Yeager et al. (2019) attributed to the increasing complexity and covert nature of bullying in late adolescence, where behaviors are more often driven by motives such as maintaining social status. Such dynamics are less pronounced in early to mid-adolescence (10–14 years) (Pichel et al., 2021). Furthermore, existing evidence indicates that bullying victimization in late adolescence tends to lead to more severe psychological and social consequences, including heightened risks of suicidal ideation (Klomek et al., 2010). These findings indicate that as bullying becomes more severe in late adolescence, the protective role of active coping strategies may diminish. Thus, we hypothesized that the moderating effect of active coping on the relationship between bullying victimization and suicidal ideation would be moderated by age cohort. Specifically,

**Hypothesis** **2.** *We expected active coping to demonstrate a reverse buffering effect among early-to-mid adolescents, whereas this effect would be weaker or non-significant among late-age adolescents.*

### 1.4. The Current Study

This study employed a cross-sectional design to test the moderating role of active coping strategies in the relationship between bullying victimization and suicidal ideation, and whether this moderating effect differed between age cohorts (early-to-middle vs. late-age adolescents). Previous studies have demonstrated that girls are more likely than boys to report suicidal ideation, possibly because they are more prone to internalizing stress and are at higher risk of developing mental health disorders. (Bauman et al., 2013; Hansen et al., 2012; Klomek et al., 2010; Liang et al., 2020; Woodhead et al., 2014). In addition, poor physical health—particularly the presence of comorbid pain conditions—has been identified as a significant risk factor for suicidal ideation (Meltzer et al., 2011), possibly because it may foster feelings of helplessness and burdensomeness, both of which have been strongly associated with suicidal ideation (van Tilburg et al., 2011; Rice & Sher, 2022). Therefore, gender and physical health status were included as covariates.

## 2. Method

### 2.1. Participants

This study employed a combination of convenience and stratified cluster sampling to recruit participants from Hubei, Jiangsu, Hunan, and Guangdong provinces in China. The target sample size was determined based on established sampling principles and previous epidemiological research on bullying among adolescents. Assuming a bullying prevalence of 15%, a 95% confidence level, and a margin of error of ±3%, the minimum required sample size under simple random sampling was calculated using the standard proportion formula (Ghardallou et al., 2024): *n* = *Z*^2^ × *p* × (1 − *p*)/*d*^2^ (*Z* = 1.96, *p* = 0.15 and *d* = 0.03), yielding an estimate of approximately 544 participants. In addition, because participants were drawn from multiple provinces and varied by school type (academically selective “key” middle and high schools versus regular schools) and school location (urban versus rural), a design effect of 2.0 was applied. After further accounting for an anticipated nonresponse rate of 10–20%, the final target sample was set at 2880 students. The final analytic sample consisted of 3227 students, exceeding the planned target and ensuring adequate statistical power and representativeness. The final sample consisted of 3227 adolescents, with a mean age of 15.03 years (SD = 1.69). Of these, 1,656 (49.8%) were male and 1671 (50.2%) were female. In terms of age cohorts, 2087 participants (64.7%) were classified as early-to-middle-aged adolescents and 1140 (35.3%) were late-age adolescents. Informed consent was obtained from all participants, their parents and the respective schools prior to data collection. The data were collected by psychology teachers of the participants and graduate students who had undergone rigorous training to ensure consistency and ethical compliance.

### 2.2. Study Procedure

Data were collected using an online questionnaire in the second half of 2019 and early 2020. For each selected class, psychology teachers first informed students about the purpose of the study, the voluntary nature of participation, and the anonymity and confidentiality of their responses. The questionnaire was administered in classroom settings during regular school hours. Postgraduate psychology students mainly assisted the psychology teachers with logistical coordination when multiple classes were surveyed at the same time, whereas psychology teachers were the primary administrators in most classes. Completion of the questionnaire took approximately 15–20 min.

### 2.3. Materials

#### 2.3.1. Bullying and Victimization

Bullying and victimization were assessed by the Chinese version of the Olweus Bully/Victim Questionnaire (OBVQ) (Olweus, 2006), revised by W. X. Zhang and Wu (1999). The scale, consisting of six items, measured the frequency and type of bullying victimization experiences. Responses were recorded on a five-point Likert scale, ranging from 0 (“never”) to 4 (“several times a week”). The bullying victimization subscale demonstrated acceptable internal consistency (Cronbach’s α = 0.78). Following the instructions of the questionnaire, participants were provided with a standardized definition of bullying, defining it as repeated negative actions by one or more students directed at another student over time. Negative actions were described as behaviors intended to hurt or upset another person, such as hitting, kicking, pushing, or saying mean or hurtful things. It was further clarified that friendly joking between classmates is not considered bullying and that occasional conflicts or fights between students of roughly equal strength are not regarded as bullying.

#### 2.3.2. Active Coping Strategy

Active coping strategies were measured using the Chinese version of the active coping subscale of Simplified Coping Style Questionnaire (Y. Xie, 1998). This subscale comprises 12 items that reflect constructive, problem-focused approaches, such as “trying to see the positive side of things” and “identifying several different ways to solve a problem.” Participants rated the frequency of their use of each strategy on a four-point Likert scale ranging from 0 (“never”) to 3 (“often”). In this study, the active coping scale demonstrated excellent internal consistency (α = 0.89).

#### 2.3.3. Suicidal Ideation

Suicidal ideation was assessed using the Chinese version of the Beck Scale for Suicide Ideation (BSI) (Li et al., 2011), originally developed by Beck et al. (1979). It assesses suicidal ideation and suicidal tendency. The first five items on this scale are suicidal ideation indicators. Following the recommendation of J. Chen et al. (2020), this study used the mean score of the first five items as the indicator of suicidal ideation. In the present study, the scale demonstrated that Cronbach’s alpha coefficient was 0.86.

#### 2.3.4. Demographic Information

Participant demographics were collected, including age, gender (coded 0 = male, 1 = female) and physical health status (coded as 1 = good, 2 = average, 3 = poor, 4 = diagnosed illness).

### 2.4. Data Analysis

IBM SPSS Statistics v29 was used for data analysis. Firstly, a hierarchical regression model was employed to examine the effects of the control variables (gender and physical health status), the independent variable (bullying victimization), the moderators (active coping strategies and age cohorts), and their interactions. Secondly, the PROCESS macro (Hayes, 2017) (http://www.afhayes.com, accessed on 20 May 2025) was used to further explore the relationship between bullying victimization and suicidal ideation under specific conditional settings.

## 3. Results

### 3.1. Visualizing Correlations Among Study Variables

**Figure 1:**
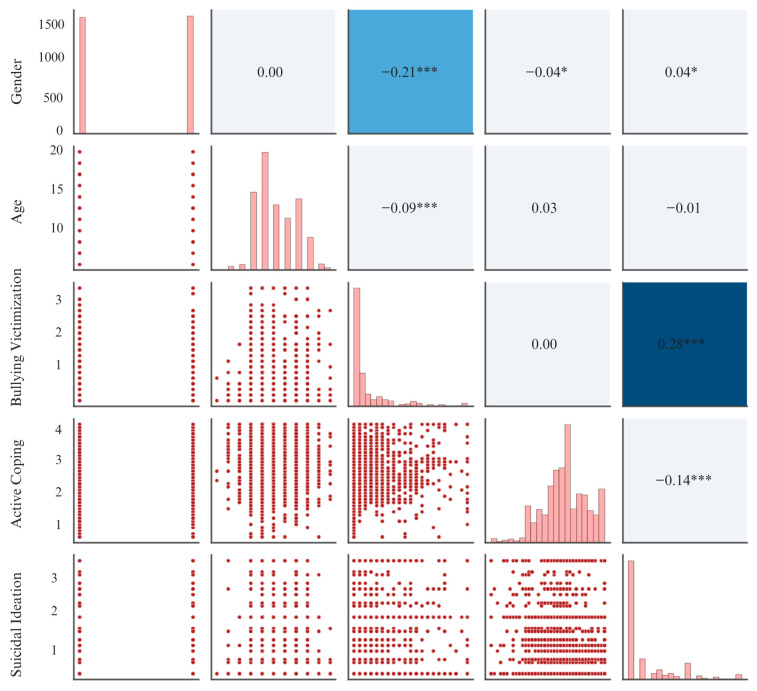
Correlation Heatmap of Key Variables. Note. Values represent Pearson correlation coefficients. Darker cell shading reflects stronger correlations. Red dots represent individual data points for each pair of variables. Asterisks indicate statistical significance: *** *p* < 0.001 and * *p* < 0.05.

As shown in Figure 1, both bullying victimization (*r* = 0.28, *p* < 0.001) and active coping (*r* = −0.14, *p* < 0.001) were significantly correlated with suicidal ideation, whereas age (*r* = −0.01, *p* = 0.462) was not significantly correlated with suicidal ideation. Moreover, Gender was a significant predictor of suicidal ideation (*r* = 0.04, *p* = 0.033), indicating that females reported higher levels of suicidal ideation than males.

### 3.2. Moderation Analysis

A hierarchical regression analysis was conducted to examine the moderating effects of active coping strategies and age cohorts on the relationship between bullying victimization and suicidal ideation. First, the control variables, including gender and physical health status, were entered into the regression equation (Model 1). Next, the interaction term bullying victimization × active coping was added to Model 1 to create Model 2. Finally, the three-way interaction term bullying victimization × active coping × age cohorts was entered into Model 2, resulting in Model 3. To ensure the robustness of the estimates, 5000 bootstrap samples were used to generate bias-corrected confidence intervals.

**Figure 2:**
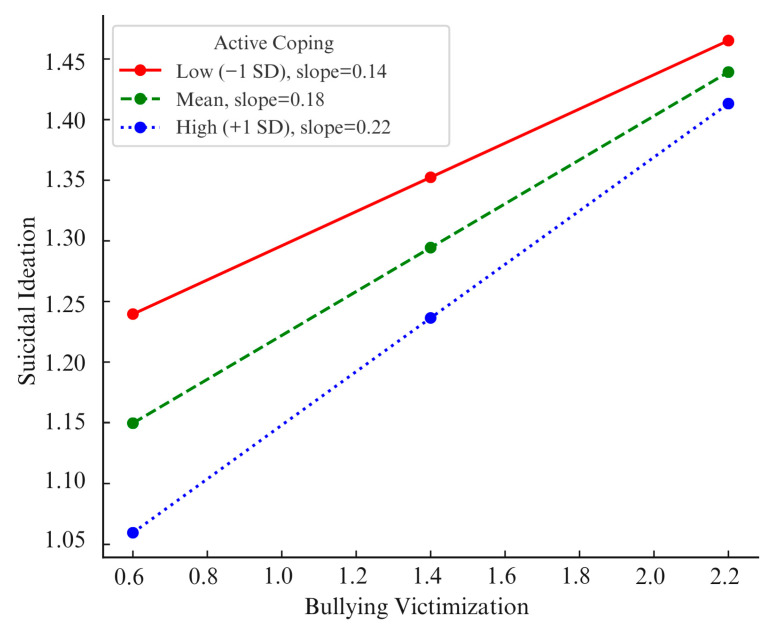
Simple Slope Plot for Moderation Analysis.

As shown in Table 1, the interaction between bullying victimization and active coping was significant (*B* = 0.03, *SE* = 0.05, *t* = 2.27, *p* = 0.023), suggesting that active coping significantly moderated the association between bullying victimization and suicidal ideation. To further probe this interaction, a simple slopes analysis was conducted. Results revealed that bullying victimization significantly predicted suicidal ideation at both high active coping condition and low condition, while the effect was weaker among adolescents with low level of active coping (*B* = 0.14, *SE* = 0.01, *t* = 10.50, *p* < 0.001) than those with high level of active coping (*B* = 0.22, *SE* = 0.01, *t* = 18.61, *p* < 0.001), revealing a reverse buffering pattern (see Figure 2). 

Notably, the three-way interaction among bullying victimization, active coping and age cohort was significant (*B* = −0.16, *SE* = 0.04, *t* = −4.38, *p* < 0.001), indicating that the moderating effect of active coping on the association between bullying victimization and suicidal ideation was further moderated by the age cohorts. To further interpret this interaction, simple slopes analyses were conducted for early-to-middle-aged adolescents and late-age adolescents, respectively.

**Figure 3:**
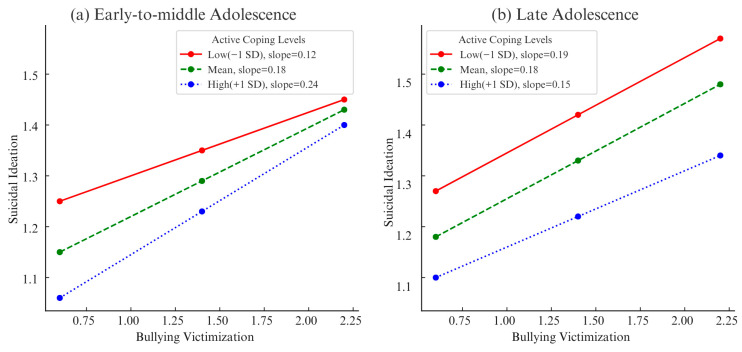
Simple Slope Plot for Moderation Analysis among Early-to-Middle and Late Adolescence.

Among early-to-middle-aged adolescents, the interaction between bullying victimization and active coping was significant (*B* = 0.10, *SE* = 0.02, *t* = 6.25, *p* < 0.001). The slope of association between bullying victimization and suicidal ideation became steeper as active coping level increased (low: *B* = 0.12, *SE* = 0.02, *t* = 7.25, *p* < 0.001; High: *B* = 0.24, *SE* = 0.01, *t* = 17.24, *p* < 0.001). As illustrated in Figure 3, among early-to-middle-aged adolescents, high levels of active coping significantly suppress suicidal ideation at low levels of bullying victimization, but once bullying victimization reaches a severe level, this protective effect is no longer significant, indicating a reverse buffering effect.

Among late-age adolescents, the interaction between bullying victimization and active coping was not significant (*B* = −0.06, *SE* = 0.03, *t* = −1.77, *p* = 0.080). 

## 4. Discussion

Consistent with previous research (Baiden & Tadeo, 2020; Bauman et al., 2013; Kerns et al., 2025; Kim et al., 2009; Kim & Leventhal, 2008), this study showed that bullying victimization was significantly positively associated with suicidal ideation. Furthermore, the results demonstrated that active coping strategies moderate this association in the opposite way; the buffering effect weakens as the severity of bullying increases. Notably, this reverse buffering effect was observed only among early-to-middle-aged adolescents, whereas no significant moderating effect was observed among late-age adolescents. These findings provide insight into the developmental nuances underlying the relationship between bullying victimization and suicidal ideation and underscore the importance of age-specific and targeted prevention and intervention efforts.

### 4.1. The Moderating Effect of Active Coping

The results showed that active coping strategies significantly moderate the relationship between bullying victimization and suicidal ideation. Specifically, active coping significantly mitigates suicidal ideation at low levels of bullying victimization. These findings align with the IMV model’s proposition that active coping serves as both TSM and MM to interrupt the shift from defeat (induced by bullying) to entrapment and then prevent entrapment from progressing to suicidal ideation.

Previous studies have highlighted the protective effects of active coping, particularly its ability to elicit positive emotions that counteract negative psychological states (Hansen et al., 2012; Lai et al., 2023; P. Sun et al., 2019). However, the present study revealed a reverse buffering effect in the relationship between bullying victimization and suicidal ideation. Specifically, active coping strategies appear to mitigate suicidal ideation under conditions of lower bullying severity, whereas this protective effect becomes non-significant as bullying becomes more severe. This likely reflects a decline in the efficacy of active coping under intense bullying. For instance, previous findings demonstrated that the effectiveness of active coping in alleviating general emotional distress wanes as bullying severity increases (Nixon et al., 2020). Our study extends this finding to a more severe psychological outcome—suicidal ideation, and suggests the reverse buffering model. From the IMV perspective, when active coping strategies fail to resolve the stressor or alleviate psychological distress, such failure may itself constitute an additional form of "defeat", making victims more prone to entrapment (Comtois et al., 2025). This helps explain why active coping remains protective under low-severity bullying conditions, while under high-severity conditions, the capacity of active coping strategies to mitigate psychological distress becomes substantially reduced. These findings remind us that, although bolstering adolescents’ active coping skills can help mitigate the suicidal ideation caused by bullying, such skills alone are insufficient in more extreme contexts. Therefore, teachers, parents and mental health professionals must step in to provide emotional support, implement practical interventions and offer professional guidance to ensure that young people do not have to face bullying’s devastating consequences in isolation.

### 4.2. The Moderating Effect of Age Cohorts

In addition to investigating the moderating role of active coping strategies in the association between bullying victimization and suicidal ideation, this study further examined differences in this moderating effect across developmental stages. Previous research suggested that bullying victimization tends to become more severe and psychologically damaging during late adolescence (Klomek et al., 2010; Pichel et al., 2021; Yeager et al., 2019). The current findings support this view and further demonstrate that the moderating effect of active coping strategies is moderated by age cohort, providing support for Hypothesis 2. Specifically, active coping significantly moderates the association between bullying victimization and suicidal ideation among early-to-middle-aged adolescents, but not among late-age adolescents. Moreover, our findings revealed that the frequency of bullying victimization declines with age, aligning with previous research (López-Castro et al., 2023). However, this reduction in prevalence does not necessarily indicate diminished psychological harm, and the frequency of bullying does not always reflect its severity (Z. Xie & Wang, 2025). In late adolescence, bullying tends to be more strategic and covert, in contrast to the more overt physical aggression or verbal provocation more commonly observed in early-to-middle adolescence (Pichel et al., 2021; Yeager et al., 2019). Furthermore, bullying in late adolescence is more likely to result in long-term psychological trauma, such as chronic depression, social withdrawal, and suicidal ideation (Klomek et al., 2010). Therefore, the absence of a significant moderating effect of active coping in this age cohort may be explained by the heightened intensity and complexity of bullying experiences, which may exceed the protective capacity of active coping. According to the IMV model, experiences of defeat or humiliation are precursors to entrapment. In early-to-middle adolescence, defeat experiences tend to be less intense and more transient; active coping strategies can effectively prevent the transition from defeat and humiliation experiences to entrapment, thereby reducing suicidal ideation. In contrast, the consequences of bullying in late adolescence are more profound and enduring, leading to severe and more persistent feelings of entrapment. Consequently, active coping strategies become less effective in mitigating suicidal ideation among late-age adolescents.

Although active coping did not moderate the association between bullying victimization and suicidal ideation among late-age adolescents, it still has a general beneficial role. Specifically, late-age adolescents with higher levels of active coping consistently reported lower levels of suicidal ideation compared to those with lower levels of coping, regardless of bullying exposure. This reflects a direct association between active coping and reduced suicidal ideation, rather than a moderating effect. Prior studies have similarly found that active coping is significantly negatively correlated with suicidal ideation in general adolescent populations, offering broad psychological protection even under specific stressor contexts (Kim et al., 2009; Kim & Leventhal, 2008; Liang et al., 2020; Meltzer et al., 2011). Therefore, fostering active coping is important for all adolescents, as it can lower the baseline risk of suicidal ideation.

### 4.3. Effects of Demographic Variables on Suicide Ideation

Regarding gender, this study revealed that girls reported a higher level of suicidal thoughts than boys, consistent with previous studies (Bauman et al., 2013; Chou et al., 2018; Hansen et al., 2012; Klomek et al., 2010; Liang et al., 2020; Woodhead et al., 2014). This gender difference may be attributed to several potential factors. On one hand, girls are more likely to internalize stress, which leads to symptoms such as anxiety, depression, and self-criticism, whereas boys are more inclined to externalize stress through behaviors such as aggression (Rosenfield, 2000). On the other hand, gender differences in the prevalence of mental health disorders may also play a role. Adolescent girls exhibit higher rates of depression and anxiety than boys—both of which are well-established risk factors for suicidal ideation (Nowotny et al., 2015).

Consistent with previous studies (Bauman et al., 2013; Meltzer et al., 2011), participants who reported better physical health in this sample exhibited significantly lower levels of suicidal ideation compared to peers with poorer physical health status. Several potential mechanisms may explain this association. First, chronic physical conditions, such as persistent pain, can impose a substantial emotional burden and foster a sense of helplessness, which may contribute to suicidal ideation independently of depressive symptoms (van Tilburg et al., 2011). Second, chronic physical illness may diminish psychological flexibility and impair future-oriented thinking, thereby undermining adolescents’ capacity to envision a meaningful future and increasing their risk of suicidal ideation (Tang & Crane, 2006). Third, reliance on others for care may foster the feeling of burdensomeness, which heightens psychological distress and intensifies feelings of worthlessness, both of which are significantly associated with elevated suicide risk (Rice & Sher, 2022). 

### 4.4. Practical Significance

The present study not only advances theoretical understanding of the effect of bullying victimization on suicidal ideation but also provides practical guidance for mitigating suicidal ideation among bullied adolescents. First, given that the severity and nature of bullying vary across developmental stages, interventions should be developmentally tailored. For early-to-middle-aged adolescents, school-based programs that cultivate active coping skills—such as problem-solving and constructive help-seeking—may be especially effective. Second, as bullying tends to be more severe and covert in late adolescence, the protective effects of active coping may diminish. Adolescents may no longer be able to cope with bullying independently. Therefore, educators and mental health professionals should adopt a more proactive role, offer individualized support and implement targeted interventions aimed at preventing crisis escalation. By translating these theory-based insights into age-appropriate strategies, educators and mental health professionals can more effectively disrupt the progression from bullying victimization to suicidal ideation.

### 4.5. Limitations and Future Directions

Although this study examined the moderating role of active coping in the relationship between bullying victimization and suicidal ideation and revealed age-related differences, there are still several limitations that remain to be addressed. 

First, the cross-sectional design restricts causal interpretation. While the findings are consistent with theoretical models positioning coping as a moderator, it remains unclear whether bullying victimization influences suicidal ideation over time or whether higher suicidal ideation leads to bullying victimization. Future research should adopt longitudinal designs to track the temporal dynamics of bullying victimization and suicidal ideation within ongoing bullying contexts.

Second, self-report measures may have introduced biases such as social desirability and recall error (Nederhof, 1985). For instance, adolescents may underreport bullying due to shame or overreport coping to appear resilient. These biases could compromise the reliability of the observed associations. Future studies should incorporate multi-informant approaches, including teacher and peer evaluations, to enhance validity.

Third, in the broader coping literature, active coping can be further divided into problem-focused and emotion-focused dimensions (Lazarus & Folkman, 1984). Because the present study employed the active coping subscale of the SCSQ, which treats active coping as a unidimensional construct, we were unable to examine whether different types of active coping differentially influence the association between bullying victimization and suicidal ideation. Future research could employ multidimensional coping measures to distinguish between problem-focused and emotion-focused active coping and investigate the unique moderating effects of active coping in each dimension.

Finally, although we examined age cohort differences in the association between bullying victimization and suicidal ideation, our measurement of victimization did not differentiate between specific types of bullying. Different forms of victimization (e.g., physical, verbal, relational, and cyberbullying) may have distinct psychological impacts. Notably, cyberbullying is more prevalent among late-age adolescents and has been linked to greater psychological harm (Bauman et al., 2013; J. K. Chen & Chen, 2020; Tran et al., 2021). Future research should explore whether active coping functions similarly across different bullying modalities.

## 5. Conclusions

This study examined the moderating role of active coping strategies in the relationship between bullying victimization and suicidal ideation. The results indicate that active coping shows a protective effect for low-to-moderate levels of bullying, buffering the association between bullying victimization and suicidal ideation. However, as bullying severity increases, this protective effect gradually weakens, reflecting a reverse buffering pattern. Furthermore, the moderating effect of active coping varies across age cohorts. Among early-to-middle-aged adolescents, active coping exhibits a reverse buffering pattern in the bullying victimization–suicidal ideation association. In contrast, among late-age adolescents, this moderating effect appears to diminish, likely due to the heightened severity and complexity of bullying at this developmental stage. These results underscore the need for educators to implement tiered, age-appropriate interventions that reduce both the incidence and severity of bullying, thereby effectively interrupting the pathway from bullying victimization to suicidal ideation.

## Figures and Tables

**Table 1:** The hierarchical regression of Bullying, Coping, and Age on Suicidal Ideation.

Predictor Variable	Outcome Variable: Suicidal Ideation					
	Model 1		Model 2		Model 3	
	*B*	*SE*	*B*	*SE*	*B*	*SE*
Gender	0.07 ***	0.02	0.08 ***	0.02	0.08 ***	0.02
Age cohort	−0.35	0.19	−0.35	0.19	−0.35	0.19
Health condition	−0.12 ***	0.02	−0.11 ***	0.02	−0.11 ***	0.02
Bullying victimization			−0.03	0.04	−0.58 ***	0.15
Active Coping			−0.20 ***	0.02	−0.37 ***	0.08
Bullying Victimization × Active Coping			0.07 ***	0.01	0.27 ***	0.05
Active Coping × Age cohort					0.14 *	0.06
Bullying Victimization × Age Cohort					0.45 ***	0.12
Bullying Victimization × Active Coping × Age cohort					−0.16 ***	0.04

*Note*. *** *p* < 0.001 and * *p* < 0.05.

## Data Availability

The data presented in this study are available on request from the corresponding author.
